# Imaging Performance of Quantitative Transmission Ultrasound

**DOI:** 10.1155/2015/454028

**Published:** 2015-10-28

**Authors:** Mark W. Lenox, James Wiskin, Matthew A. Lewis, Stephen Darrouzet, David Borup, Scott Hsieh

**Affiliations:** ^1^QT Ultrasound, LLC, Novato, CA 94949, USA; ^2^Department of Radiology, University of Texas Southwestern Medical Center, Dallas, TX 75390, USA

## Abstract

Quantitative Transmission Ultrasound (QTUS) is a tomographic transmission ultrasound modality that is capable of generating 3D speed-of-sound maps of objects in the field of view. It performs this measurement by propagating a plane wave through the medium from a transmitter on one side of a water tank to a high resolution receiver on the opposite side. This information is then used via inverse scattering to compute a speed map. In addition, the presence of reflection transducers allows the creation of a high resolution, spatially compounded reflection map that is natively coregistered to the speed map. A prototype QTUS system was evaluated for measurement and geometric accuracy as well as for the ability to correctly determine speed of sound.

## 1. Introduction

**Figure 1:**
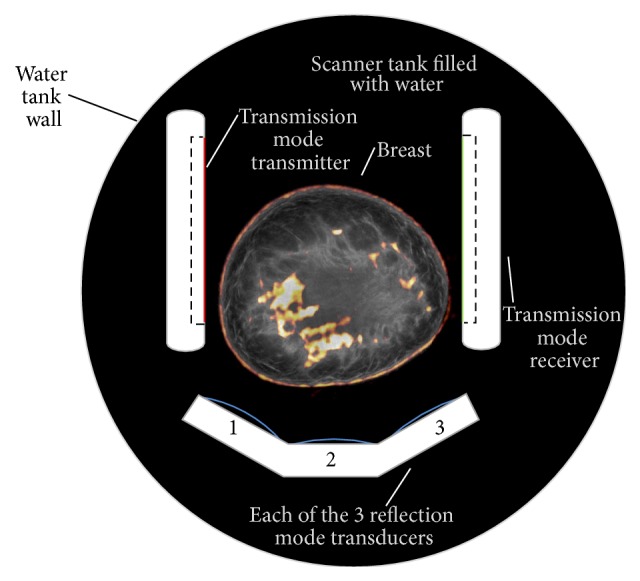
System geometry.

Quantitative Transmission Ultrasound (QTUS) is an imaging modality based on tomographic techniques extended to ultrasound. In such a system, images are generated using both reflection and transmission techniques. While transmission ultrasound has been investigated as an adjunct to mammography for quite some time [1, 2], recent developments in hardware and imaging algorithms have enabled marked improvements in spatial resolution and clinical utility. Physically, a transmitter and receiver pair is colocated with multiple transducers with various focal lengths in a U-shaped arrangement as shown in Figure 1.

This type of device has been shown to work well in breast imaging applications [3–6]. Early work to characterize systems of this nature has been performed [7], but much remains to be done to move toward reproducible methods that can be applied to all systems of this type. No NEMA standards have been developed for 3D tomographic ultrasound methods. There are a number of characteristics of QTUS systems that make them neither entirely tomographic in the normal sense, nor standard B-mode, and this represents a challenge to properly evaluate their performance. For example, typical ultrasound characteristics, like speckle, are eliminated by the B-mode compounding, so alternatives must be developed to give potential users a better grasp of what contrast to noise type characteristics might mean in a clinical setting. Furthermore, shadowing is also severely depressed and this makes the measurement of system resolution in plane a true 2D problem instead of a 1D problem, even against a hard fast target. This paper proposes methods to evaluate such systems in ways that would be familiar to those working in other modalities and presents preliminary findings for such a system.

In transmission mode, the transmitter emits a plane wave that is received by the receiver. In this case, the receiver is a 1536 element PZT array with custom data acquisition electronics package that supports real-time RF data acquisition at rates of 33.3 Ms/s at 14 bits per sample. Multiple acquisitions at frequencies ranging from 300 kHz to 1.5 MHz are acquired for 180 angles as the U-channel is rotated around the subject. Once acquired, the projection information is reconstructed using nonlinear inverse scattering in 3D [3–6]. The result of this reconstruction is a quantitative volume map of speed of sound (measured at 1.5 MHz), with units of meters per second (m/s), and attenuation with units of dB/m/MHz.

In reflection mode, each of the three reflection transducers (4 MHz center frequency) with different focal lengths are alternately fired between transmission measurements in a B-mode acquisition. The resulting images (60 per transducer) are compounded together and corrected for refraction using the speed map computed in the transmission phase. This compounding produces a nonquantitative image that is proportional to impedance mismatch, referred to simply as reflection units (RUs). Since impedance mismatch for ultrasound waves indicates a change in tissue type, these images have high resolution and are very instructive of anatomical reference. Due to the nature of this type of compounding, speckle is dramatically reduced, but the resulting image can be read very much like a traditional B-mode image.

The end result of each scan is a 3D volume of three different types, speed, attenuation, and reflection. These image stacks are precisely coregistered since they were acquired at the same time and can be put together to form a 3D view of the object in the field of view (FOV). The 3D nature of the device allows the evaluation of both 2D qualities, like dimension and relative placement, as well as 3D physical qualities, such as the volume of a structure and the quantitative evaluation of the speed of sound within the entirety of that volume. To date, internal research studies have concentrated on the use of speed-of-sound information and this paper will concentrate on the accuracies of those measurements; however, future studies are planned to include analysis of attenuation information.

## 2. Materials and Methods

In this experiment, the current generation QT Ultrasound system (QT Ultrasound LLC, Novato, CA) was evaluated in both transmission and reflection modes for geometric accuracy and uniformity over the field of view and for quantitative accuracy of the speed-of-sound maps. Several phantoms were constructed to test speed-of-sound quantitative accuracy, geometric accuracy, contrast resolution, contrast to noise ratio, and uniformity. In addition, laboratory experiments were performed to independently evaluate the expected speed values of the various materials used to construct the phantoms.

### 2.1. Speed of Sound

A phantom was constructed and evaluated using the QTUS system as well as analytic methods. This particular phantom contains inclusions 15 mm in diameter with different speeds to simulate cyst and solid lesion pathologies. It is designed to mimic soft tissue regions such as breast as there are no high speed structures to simulate bone.

All materials are custom formulated polyurethanes designed for a specific speed with the high speed material provided by Yezitronix (Yezitronix Group Inc., Canada) and the low speed and background materials were supplied by Conversion Technology (Conversion Technology Inc., Boulder, USA). Samples of each material in the speed phantom were provided separately in the form of a 2′′ diameter cylinder with a 1′′ thickness. These samples could be independently evaluated by measuring the change in signal phase when the sample is placed between two 5 MHz pistons, relative to the water path signal phase with the sample removed.

### 2.2. Spatial Measurements

Tests are included to measure both the spatial resolution of the device and the spatial accuracy when measuring distances between varieties of object sizes across the field of view.

Spatial resolution is patterned after the NEMA standards for other tomographic modalities [8], with measurements in the center as well as at varying radii from the center in the transaxial direction as well as a slice thickness definition. Unlike modalities such as PET, where it is physically possible to build point sources that are substantially smaller than the intrinsic resolution and measure the system response directly as an impulse response, QTUS has very high theoretical resolution and thus determining the intrinsic resolution from the impulse response of the system is problematic. Previous work has relied on a single step edge method [7] to perform this evaluation; however, it is possible to bias that measurement in the direction of the step and it also requires exceptionally small voxels to properly compute the derivative of the gradient function. In order to get around these limitations we propose to evaluate the square step response and remove the step width using the sum of squares approach. This approach involves measuring the gradient in two opposing directions to eliminate bias. In this method, the measured response, *R*_meas_, is the convolution of the intrinsic resolution *R*_int_ and the actual object size *S*_obj_. Since these represent Gaussian functions, then their relationship can be expressed as the sum of squares:

(1) $$\begin{array}{c} {R_{int}}^{2}+{S_{\text{obj}}}^{2}={R_{\mathrm{meas}}}^{2}. \end{array}$$

Since the spread of the object is precisely determined by the actual known size, determination of *R*_int_ becomes straightforward. With this method, the intrinsic resolution can be evaluated without dependence on the ability to construct a particular sized target to evaluate the system response function. It is potentially possible to bias this measurement by choosing an object of a specific size, so it is important to specify completely the size of the object used when specifying the resolution.

For this evaluation in transmission mode, a spatial measurement phantom is used. This phantom consists of styrene monofilament stretched between two styrene plates arrayed in a nautilus spiral starting at the center. The diameter of the monofilament can be easily changed depending on the type of imaging performed. We choose 608 microns for speed measurements to achieve the necessary contrast. As the ability to perform full resolution measurements improves, it is likely that this measurement standard will change, and the monofilament will get smaller. Full width at half maximum (FWHM) width measurements are made in both the *X* and *Y* dimensions as shown in Figure 4, measured by linearly interpolating known data points on each side [8], and then deconvolved from the object size using (1). By choosing a nautilus spiral, it is possible to get a relatively clear view of all filaments with a minimum of interference. Since transmission measurements are made in a line through the object, a thin cylindrical target performs well and does not require precise vertical positioning to get consistent results.

It is important to consider that resolution enhancing methods like point-spread-function modeling can sharpen the resulting clinical images; however, the use of PSF modeling in the reconstruction will interfere with these measurement methods. The desired result of this measurement is the intrinsic resolution of the system which could then be used for PSF modeling and further improvements in clinical utility. Thus, for the purposes of this paper, all PSF modeling is disabled within the reconstruction.

Measurement of reflection spatial resolution requires the use of spherical targets because the geometry of a line crossing the beam causes reflections that can vary considerably with the initial angle of incidence of the beam to the monofilament line and this makes an unbiased measurement very difficult. One family of phantoms developed in collaboration with colleagues at UT Southwestern Medical Center is comprised of a gelatin outer layer with an inner core comprised of the synthetic clay Laponite XLG (BYK Additives, Gonzales, TX). Using tweezers, soda lime glass beads with nominal diameter of 500 micrometers (Cospheric LLC, Santa Barbara, CA) were manually placed in a plane in the middle of the Laponite at coordinates (0,0), (0.8660,0.500), (1.4141, −1,4142), and (−1.500, −2.5981) cm. The gelatin skin is formed in a 90 mm inner diameter disposable 8 oz cup (http://www.us.huhtamaki.com/) using a glass beaker coated with nonstick spray oil to form the inner chamber. The gelatin powder (Type B from bovine skin, Sigma Aldrich) is mixed in a 1 : 5 ratio with deionized and degassed water in a larger beaker and heated under mild stirring to at least 35 Celsius. After 30 minutes, the beaker is further degassed, and remaining bubbles and detritus on the surface are removed with suction. As the gelatin cools to below 32 Celsius, the gelatin stock is titrated with formalin (Sigma Aldrich) at 1.4 mL per 40 mL of water. This amount of formalin is known to cross-link gelatin such that the melting temperature is increased above the 31 Celsius temperature of the ultrasound tomography water bath [9].

The inner chamber is filled with Laponite XLG, a magnesium silicate gel with baseline speed of sound below 1500 m/s [10]. The Laponite powder is rapidly mixed with water under vigorous stirring. After some time, the mixture becomes clear, at which point the stock material is treated similarly for bubble removal. The inner chamber of the phantom is filled with Laponite to the level where inclusions are to be added. After the soda lime glass beads are placed, the partially assembled phantom is cooled in a refrigerator for 10 minutes so that the beads adhere to the Laponite. Then they are covered with additional Laponite material. The phantom is completed with a second formulation of gelatin to pour a top for the phantom. Some changes in the Laponite compartment size are observed in the days following construction, presumably due to equalization of water content.

To measure slice thickness, a dual inverted comb phantom is used. A series of 2 mm diameter styrene rods are positioned linearly with a 0.5/10 mm gradient such that the imaging plane covers both combs as in Figure 5. The slice thickness is defined by the thickest point where it is possible to resolve the ends of one of both combs.

To compute slice thickness, an image plane is selected that cuts across both of the comb gradients perpendicular to the rods. The upper level and lower level included in the slice are noted by observing which rods are present in the image. As the rods subside from the plane at a rate of 0.5 mm per 10.0 mm a linear relationship is established to interpolate submillimeter changes in the plane boundaries. The difference in the plane boundaries is the slice thickness.

### 2.3. Uniformity

Uniformity for speed measurements is patterned after the other NEMA uniformity performance measurement protocols [8]. A uniform phantom (8 cm diameter, 4 cm axial extent) made of polyurethane is scanned, and a volume region of interest (ROI) is made 2 mm inside of the outer edge for all planes that are completely inclusive. The polyurethane has a low uniform speed of 1430 m/s, and, since there are no changes in material, there should be very low reflectivity throughout the object. It is important that there be no gasses present as air bubbles can significantly alter the measurement. It is recommended that the phantom be soaked in the water tank for at least an hour prior to this test to assure uniform temperature distribution and absorption of any exterior bubbles.

We define that the uniformity *U* of the region is expressed as a percentage of the standard deviation *σ*_roi_ divided by the mean value *μ*_roi_ of the region of interest:

(2) $$\begin{array}{c} U=\frac{\sigma_{\text{roi}}}{\mu_{\text{roi}}}*100. \end{array}$$

Uniformity for reflection images is not well defined. Since reflection is a measure of impedance mismatch, a truly anechoic cylinder volume will have a mean value of zero. This makes (2) unstable in this case. Further work in measurement of reflection uniformity is needed but is beyond the scope of this paper.

### 2.4. Contrast to Noise Ratio and Contrast Resolution

Contrast to noise ratio (CNR) expresses the potential to detect an object against a background that contains a noise component. In this case, we define CNR as the difference between a region containing the object and the background, divided by the standard deviation of the background:

(3) $$\begin{array}{c} \text{CNR}=\frac{S_{\text{obj}}-S_{\text{bkd}}}{\delta_{\text{bkd}}}. \end{array}$$

A contrast phantom, made up of styrene rods placed in a circle, shown in Figure 6, is used to evaluate CNR against multiple targets at the same time. The contrast resolution (CR) of a device is defined to be the point in which the CNR no longer allows identification of an object smaller than that dimension.

## 3. Discussion

### 3.1. Results

#### 3.1.1. Speed-of-Sound Accuracy

**Figure 2:**
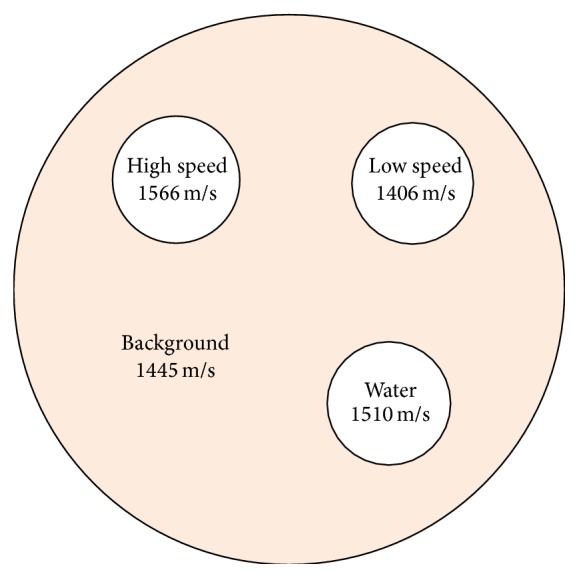
Speed phantom specifications.

**Figure 3:**
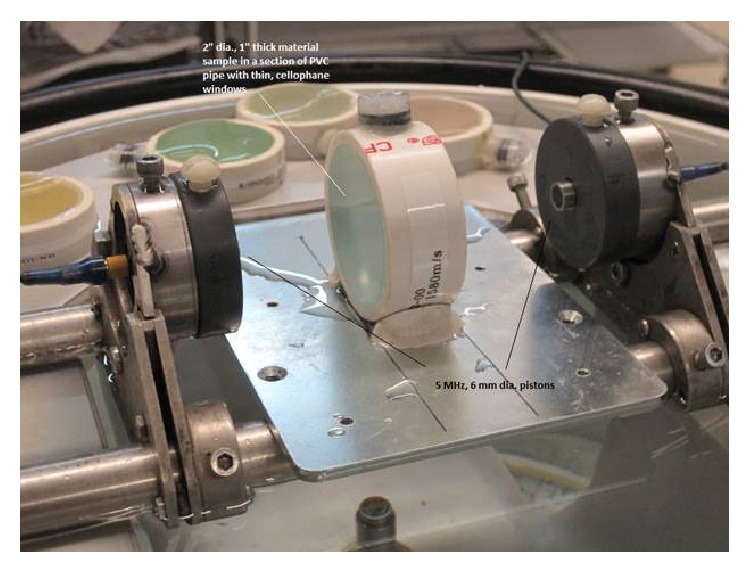
Experimental setup. Two, colinear, 5 MHz, 6 mm diameter piston transducers are mounted 100 mm apart. The 2′′ diameter, 1′′ thick material sample is placed between them normal to the beam axis.

**Figure 4:**
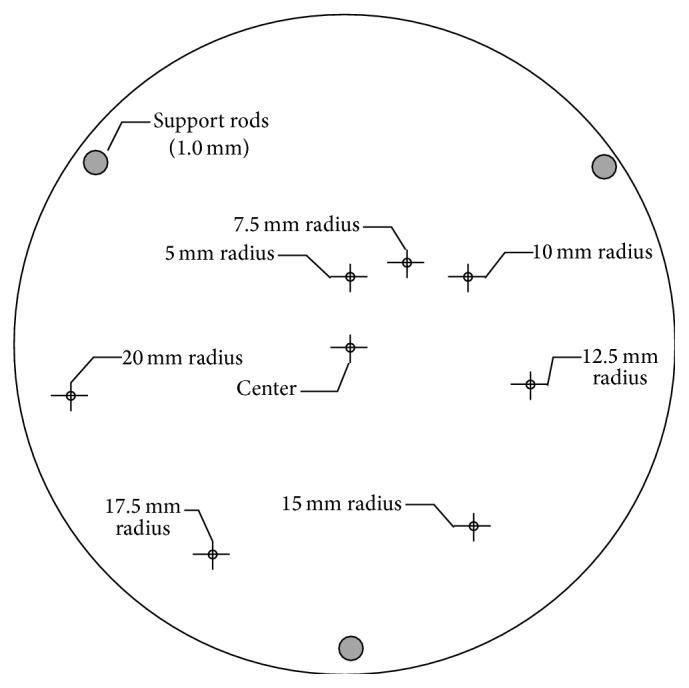
Transmission spatial resolution phantom dimensions.

**Figure 5:**
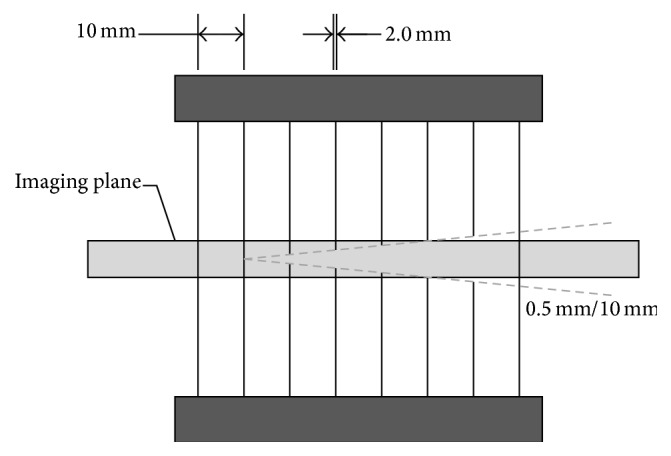
Slice thickness phantom. Dual inverted comb gradients locate the upper and lower edge of the imaging plane to determine actual slice thickness.

**Figure 6:**
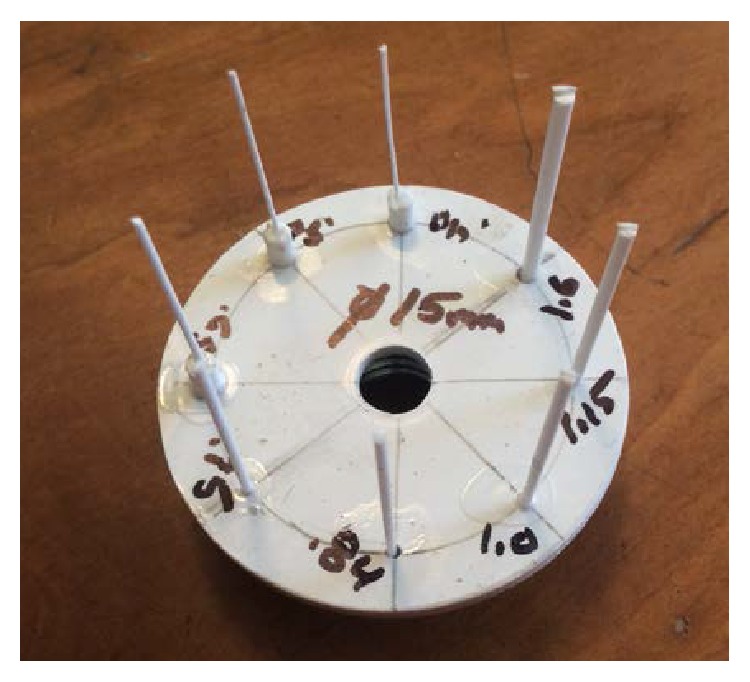
Contrast phantom.

Speed-of-sound measurements were performed on the speed phantom shown in Figure 2. Actual speeds were measured with the piston setup shown in Figure 3. Error was computed as difference from the actual values measured with the pistons and expressed as the percentage of the actual value as well as in standard deviations from the region-of-interest (ROI) measurement. The percentage of actual error provides the overall magnitude of the possible error. The error expressed in standard deviations provides a measure of accuracy relative to the expected accuracy within the ROI. Values less than 1.0 indicate that the error is less than the measured noise in the ROI. As reported in Table 1, in the range of typical tissue values (water speed and faster), the system was able to accurately determine speed of sound within 1% of the actual value in all cases.

#### 3.1.2. Transmission Resolution Measurements

**Figure 7:**
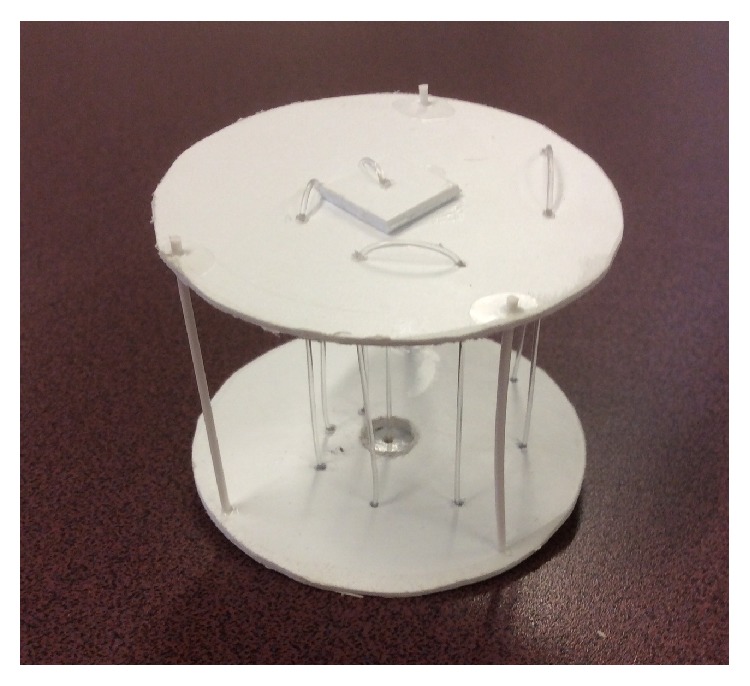
Nautilus phantom.

Transmission measurements were performed on the nautilus phantom using 608-micron styrene monofilament submerged in water. As constructed, the nautilus phantom is shown in Figure 7.

**Figure 8:**
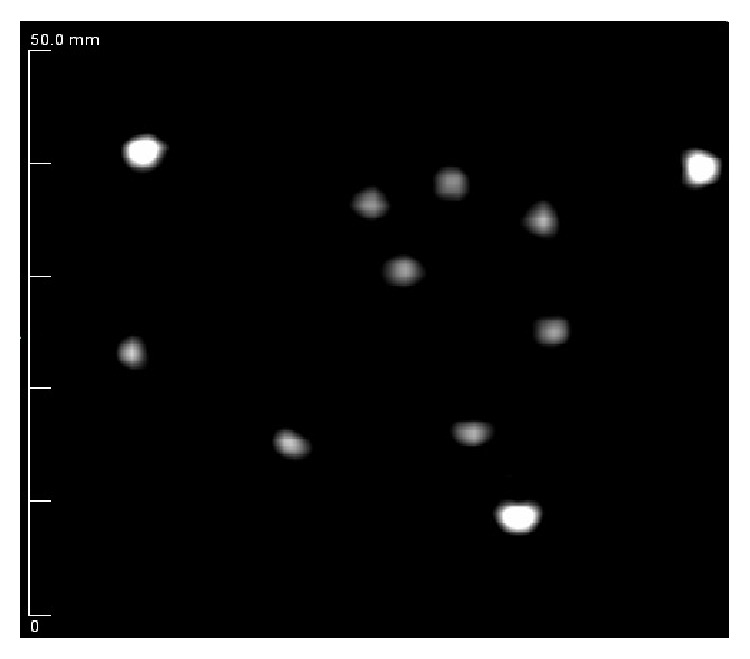
Speed-of-sound image of nautilus phantom showing 608-micron monofilament structures window leveled to show maximum contrast against a water background (grayscale in m/s).

Good contrast was achieved against the water background as shown in Figure 8, allowing accurate measurements at all radius values. Measured FWHM values were corrected with (1) and the resulting intrinsic resolution values are given in Table 2.

FWHM values were relatively flat across the FOV, varying from 1.870 mm to 2.490 mm with an average of 2.335 mm in both *X* and *Y*. Measurements performed with the dual inverted comb phantom yielded an actual slice thickness of 2 mm in transmission (speed-of-sound) mode.

#### 3.1.3. Reflection Resolution Measurements

The reflection resolution phantom with embedded 500-micron beads was scanned. The results are shown in Table 3.

Resolution results were relatively flat across the field of view with the exception of the exact center where they were slightly degraded as would be expected due to additional scattering at depth and residual refraction effects. Average resolution across the entire FOV was 543 microns in the *X* direction and 557 microns in the *Y* direction. However, average resolution of all areas except the exact center is significantly better at 453 microns in the *X* direction and 482 microns in the *Y* direction. As this represents the field of view most commonly used in normal observations, this is the level of performance that should be expected in general operation. Measurements performed with the dual inverted comb phantom yielded a slice thickness of 6 mm in reflection mode.

#### 3.1.4. Uniformity

**Figure 9:**
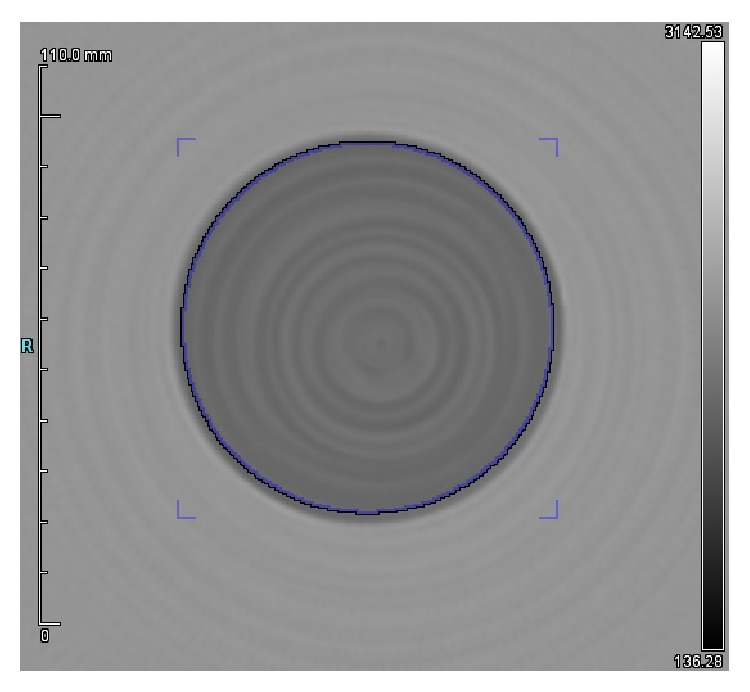
Speed-of-sound image of a uniform phantom (grayscale in m/s, max 3142 m/s, min 136 m/s).

An 80 mm uniform phantom made of T2039-6634 polyurethane (Conversion Technology Inc., Boulder, CO, USA) was scanned in both reflection and transmission modes as shown in Figure 9.

Overall, the mean speed of a region 2 mm inside of the outside edge of the phantom was measured to be 1401.9 m/s with a standard deviation of 44.1. According to (2), this yields a uniformity of 3.1%.

#### 3.1.5. Contrast to Noise Ratio and Contrast Resolution

**Figure 10:**
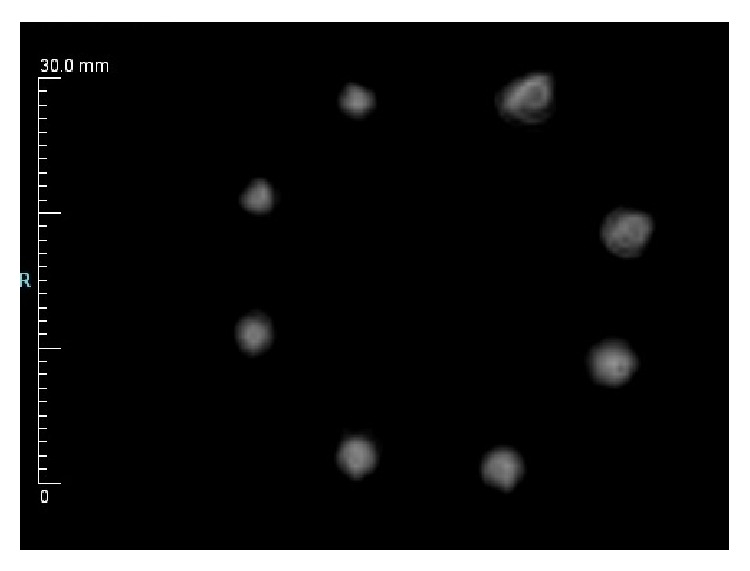
Contrast resolution reflection results.

The contrast resolution phantom was scanned in both reflection and transmission modes. Reflection mode, shown in Figure 10, yields excellent results from 1.6 mm down to 400 microns. Performance is essentially flat down to 400 microns for these high speed targets against a water background. This result suggests a contrast resolution better than 400 microns in reflection mode. Reflection mode measures impedance mismatch, so since the material in each rod is the same, the mismatch against water should also be the same and all intensities should match. The larger rods, 1.6 mm and 1.15 mm, show slight nonuniformities due to the 10-degree angle of incidence that the reflection transducers have against the vertical rods.

**Figure 11:**
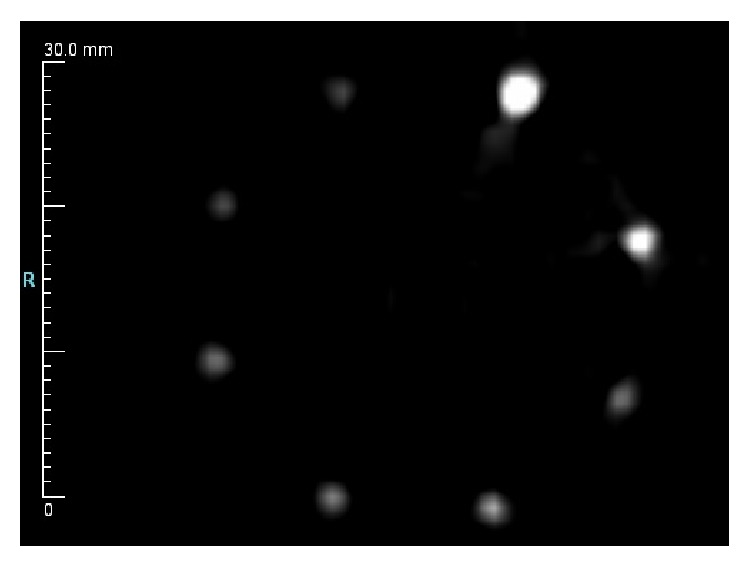
Contrast resolution speed results window leveled to highlight maximum available contrast.

Contrast resolution in transmission mode is shown in Figure 11. All rods are visible from 1.6 mm down to 0.4 mm with expected degradation in signal strength/contrast as the object size decreases. The actual speed of the styrene plastic samples measured with the previously described piston method on a large sample indicates 2000 m/s. The largest sample size of 1.6 mm diameter indicates a speed of 1949 m/s, slowly degrading to 1574 m/s for the 400-micron pin due to partial volume effects. These results are summarized in Table 4.

Contrast resolution performance overall indicates that detectability of dense structures should be very good, even in the submillimeter range.

#### 3.1.6. Contrast to Noise Ratio

**Figure 12:**
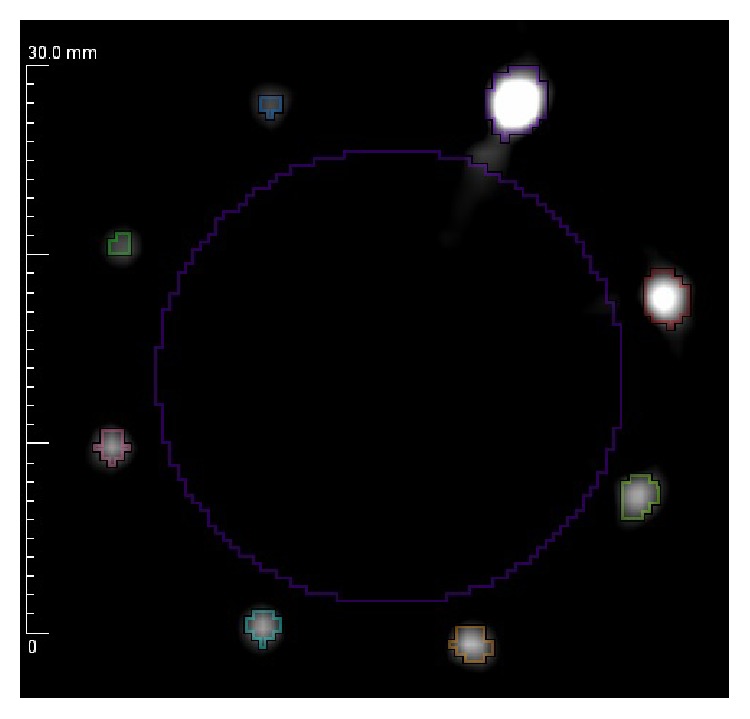
Contrast to noise ratio ROI measurement locations (grayscale in m/s).

The design of the CNR phantom with 8 separate targets integrated allows a single scan to yield 8 individual measurements under exactly the same conditions (i.e., temperature). Contrast to noise ratio was measured by drawing regions surrounding the rods with an isocontour. All regions used the same arbitrary lower limit, with no effective upper limit so that the mean value can be compared directly. The background region was chosen to be inside the objects as shown in Figure 12.

After applying (3), CNR values were computed from 1.4 mm to 0.4 mm for both speed of sound and reflection information with results given in Table 4.

The actual speed of styrene was measured using the dual-piston method previously described as 2002 m/s. In speed mode, the CNR steadily deteriorates as the size of the object becomes smaller, but it remains better than 10 : 1 even on objects as small as 400 microns. As would be expected, the algorithm underestimates the actual values as the size gets smaller and partial volume effects come into play.

Reflection performs very well across the entire range, staying essentially flat down to 400 microns. This result is expected and in fact is one of the more powerful applications of ultrasound. Under most conditions, objects will cause a discernable reflection between adjacent neighboring scatterers as long as their size is greater than half the wavelength of the wave. In this case, at 4 MHz, the wavelength is approximately 0.382 mm, so, theoretically, objects should provide signal at 0.191 mm. This theoretical minimum is smaller than the 0.40 mm objects used in this test.

## 4. Conclusions

It has been shown in the literature that ultrasound can be used to evaluate the pathology of lesions in a variety of cancers [11]. The ability to perform this evaluation is largely due to both the fact that the speed of sound is directly related to the bulk and shear modulus of the material and the fact that the structural changes to the tissue that arise from various pathologies affect the bulk modulus. This relationship suggests that direct speed-of-sound measurements have positive potential to discriminate various pathologies, including those that exhibit some type of calcification similar to elastography.

QTUS provides a stable measure of both geometry and speed of sound on objects as small as 0.4 mm in diameter, and possibly smaller. Contrast resolution and CNR experiments, in particular, show considerable promise in reflection as well as transmission modes of operation when high speed contrast targets are present. These conditions are very similar to conditions present when imaging calcifications based on Calcium Oxalate and Calcium Hydroxyapatite, the typical pathologies present in calcified breast lesions, and Ductile Carcinoma In Situ (DCIS) [12]. In addition, spatial resolution and accuracy are good enough to encourage accurate location and biopsy of extremely small lesions.

As a first step, future work should include more phantom studies with smaller structures to fully define the limits of the technology. In addition to that, work should include clinical trials to perform a proper receiver operating characteristic (ROC) analysis of a QT Ultrasound system in evaluating calcifications.

## Figures and Tables

**Table 1:** Speed-of-sound accuracy against the speed phantom measured with twin pistons.

Area	Mean (M/S)	SD (M/S)	Uniformity (%)	Actual (M/S)	Error (M/S)	Error (% actual)	Error (SD)
High	1571.9	18.1	1.15%	1566	5.9	0.37%	0.33
Low	1418.8	5.69	0.40%	1406	12.8	0.91%	2.25
Water	1508.6	7.09	0.46%	1510	1.4	0.09%	0.20
Background	1447.9	6.24	0.43%	1445	2.9	0.02%	0.46

**Table 2:** Transmission resolution measurements.

Position	*X* FWHM (mm)	*Y* FWHM (mm)
Center	2.11	2.06
5 mm	2.45	2.36
7.5 mm	2.18	2.40
10 mm	2.13	2.49
12.5 mm	2.31	2.03
15 mm	2.41	1.94
17.5 mm	2.20	2.10
20 mm	1.87	2.32

**Table 3:** Reflection resolution measurements.

Position	*X* FWHM (mm)	*Y* FWHM (mm)
Center	0.771	0.751
10 mm	0.346	0.543
20 mm	0.536	0.373
30 mm	0.478	0.531

**Table 4:** Speed and reflection CNR.

Object	Speed CNR	Reflection CNR	Speed m/s
1.6 mm	28.4	2.7	1949
1.15 mm	21.5	3.6	1786
1.0 mm	15.5	5.9	1657
0.84 mm	15.7	3.8	1626
0.75 mm	14.4	4.5	1611
0.65 mm	13.5	4.2	1610
0.50 mm	10.8	4.3	1582
0.40 mm	10.2	4.0	1574
